# Combined model of topside ionosphere and plasmasphere derived from radio-occultation and Van Allen Probes data

**DOI:** 10.1038/s41598-022-13302-1

**Published:** 2022-06-13

**Authors:** Fabricio S. Prol, Artem G. Smirnov, M. Mainul Hoque, Yuri Y. Shprits

**Affiliations:** 1 German Aerospace Center (DLR), Institute for Solar-Terrestrial Physics, Neustrelitz, 17235 Germany; 2 Finnish Geospatial Research Institute (FGI), Department of Navigation and Positioning, National Land Survey of Finland (NLS), Kirkkonummi, 02431 Finland; 3 Helmholtz Centre Potsdam (GFZ), German Research Centre for Geosciences, Potsdam, Germany; 4 University of Potsdam, Institute of Physics and Astronomy, Potsdam, Germany; 5 Department of Earth, Planetary and Space Sciences, University of California Los Angeles (UCLA), Los Angeles, USA

**Keywords:** Atmospheric science, Space physics

## Abstract

In the last years, electron density profile functions characterized by a linear dependence on the scale height showed good results when approximating the topside ionosphere. The performance above 800 km, however, is not yet well investigated. This study investigates the capability of the semi-Epstein functions to represent electron density profiles from the peak height up to 20,000 km. Electron density observations recorded by the Van Allen Probes were used to resolve the scale height dependence in the plasmasphere. It was found that the linear dependence of the scale height in the topside ionosphere cannot be directly used to extrapolate profiles above 800 km. We find that the dependence of scale heights on altitude is quadratic in the plasmasphere. A statistical model of the scale heights is therefore proposed. After combining the topside ionosphere and plasmasphere by a unified model, we have obtained good estimations not only in the profile shapes, but also in the Total Electron Content magnitude and distributions when compared to actual measurements from 2013, 2014, 2016 and 2017. Our investigation shows that Van Allen Probes can be merged to radio-occultation data to properly represent the upper ionosphere and plasmasphere by means of a semi-Epstein function.

## Introduction

The topside ionosphere has been investigated since the beginning of the development of electron density models to describe the electron density dependence with the altitude. First attempts to represent the topside ionosphere was conducted to extrapolate ionosonde profiles to altitudes above the peak height. Early work shows that a single-layer Chapman function^1^ can successfully describe the exponential decay of electron density at the topside. However, with the development of new techniques to observe the upper ionosphere, more robust mathematical functions were developed, such as exponential^2^, parabolic (or sech-squared)^3^, Semi-Epstein^4–7^ and further versions of Chapman^8,9^ functions.

During the 1960s and 1970s, observations from the topside sounders on board the International Satellite for Ionospheric Studies (ISIS) and Allouete satellites have provided good specifications of the topside, being used as input data to models such as the International Reference Ionosphere (IRI)^10^ and NeQuick^11^. Based on ISIS observations, Fonda et al.^12^ presented a comparison between distinct analytical functions and found that Chapman functions best describes the topside ionosphere. Their experiments were conducted using single-layer functions and suggestions were given to the use of multi-layer approaches for further improvements. Indeed, Vary-Chap functions, which model the topside with a varying scale height, have become a default option to improve the topside profile representation of IRI^13,14^.

Lately, investigations of suitable topside functions have gained increased attention due to the outstanding data provided by Radio-Occultation (RO) retrievals from Global Navigation Satellite Systems (GNSS) receivers onboard Low Earth Orbit (LEO) satellites. The configuration of the LEO and GNSS satellite orbits produces continuous data of the topside electron density with global coverage. Based on the GNSS-RO retrievals, Olivares-Pulido et al.^15^ demonstrated that the Vary-Chap function with a linear scale height is very useful to represent the topside RO electron density profiles. Additionally, Hernández-Pajares et al.^16^ have shown comprehensive results in the extrapolation performance of the linear scale height in the region above 500 km of the ionosphere. Building on such works, Prol et al.^17,18^ have developed a 4D ionospheric model and presented that the topside is well described and predicted by the linear Vary-Chap functions. Recently, Pignalberi et al.^19^ have used a semi-Epstein function to represent the linear scale height. As a result, linear scale height was in excellent agreement with observational data and theoretical physics. Therefore, up to now, it is known that the linear scale height, by Vary-Chap or semi-Epstein functions, well accommodates to ionosphere observations from the peak height (hmF2) up to around 800 km.

Above the ionospheric boundaries, in the plasmasphere, a large and growing body of literature has been devoted to understanding the dynamics and morphology of the region. According to D. L. Carpenter^20^, the existence of the plasmasphere was first deduced by L. R. Owen Storey^21^, showing that whistler signals follow long paths between the hemispheres through a plasma cloud that surrounds the Earth. Thanks to advances in the probes of the Earth’s plasma envelope in the early 1960s, whistler and rocket measurements were independently collected by Carpenter^22^ in the USA and by Gringauz^23^ in the Soviet Union to detect a geomagnetic-field-aligned boundary that represents the edges of the plasmasphere, i.e., the plasmapause. In the many years of consequent study, diverse quantitative analytical models have been developed. Valuable models of the plasmasphere are the Carpenter and Anderson model^24^, IZMIRAN plasmasphere model^25^, University of Graz electron density global ionospheric model (NeUoG^26^), global core plasma model (GCPM^27^), O’Brian and Moldwi model^28^, Pierrard and Stegen model^29^, and the Neutrelitz electron density model (NEDM^30^). Empirical models of the plasmasphere have greatly improved in the recent years and are now capable of reproducing complicated dynamics, including the plasmaspheric erosion and plume formation and evolution (e.g., Zhelavskaya et al.^31^).

Although several studies have been conducted to model the plasmasphere and topside ionosphere, there is, yet, a large spatial gap, spanning from approximately 800 km up to several thousand km where only very few data points are available. Finding a suitable connection between the ionospheric and plasmaspheric models therefore remains a significant challenge, with a few works (e.g., Třísková et al.^32^) addressing solutions to the problem. Several recent studies have indicated that the existing models do not provide accurate electron density values at higher altitudes. For instance, Cherniak and Zakharenkova^33^ points out that IRI-Plas^34^ and NeQuick^11^ needs to be essentially improved above the F2 layer peak (hmF2) for better characterization of the topside Total Electron Content (TEC). Additionally, Kashcheyev and Nava^35^ and Prol et al.^36^ show that NeQuick is systematically predicting lower TEC values in comparison to satellite-based TEC measurements.

In the present study, our main motivation is to find a suitable scale height function to describe the electron density from hmF2 up to the GNSS orbit height. In this regard, we have revisited several topside measurements to check whether this data can be accurately approximated using linear scale functions. An investigation was carried out to analyze the possibilities of representing the electron density profiles based on GNSS-RO topside data, which provides profiles distributions from hmF2 up to 800 km. Van Allen Probes in-situ measurements were used to describe the electron density values above 800 km. As a result, we proposed a scale height function using RO and Van Allen Probe data to represent a combined model of the ionosphere and plasmasphere.

## Dataset

The present study is performed with Van Allen Probe measurements to provide electron density measurements at altitudes up to 5.8 Earth radii. The electron density data was defined from dynamic spectrograms by Zhelavskaya et al.^37^ using the algorithm NURD (Neural-network-based Upper hybrid Resonance Determination). RO-Ne profiles and TEC values are retrieved from GNSS receivers onboard the Formosat mission 3 / Constellation Observing System for Meteorology, Ionosphere, and Climate (FORMOSAT-3/COSMIC, or just COSMIC). The COSMIC satellite mission provides electron density profiles obtained by RO techniques from the topside ionosphere, while the COSMIC onborad receivers for precise orbit determination (POD) provides TEC values above the LEO orbit height. We have used level 1 product, namely podTec, to obtain TEC values and level 2 product, namely ionPrf, to obtain RO-Ne profiles. The dataset was processed by the University Corporation for Atmospheric Research (UCAR). A detailed view of the COSMIC TEC quality is provided by Yue et al.^38^ and details on the process to derive electron density values of the ionPrf products are provided by Schreiner et al.^39^. The main source of errors in the retrievals is related to TEC precision, spherical symmetry assumptions, straight-line signal approximation, and miss-modeling of the contribution of the electron content above the LEO satellite. The latter source of error usually requires to be modeled by a plasmaspheric climate model or somehow extrapolated. Although the plasmaspheric contribution should be accounted in a reasonable way, Aragon Angel et al.^40^ concluded that the relative accuracy of the profiles only improved by about 2% when analyzing distinct extrapolation techniques compared to calibrated ionosonde data.

## Effective scale height obtained by the semi-epstein function

The semi-Epstein formulation is used in this work to represent the electron density at the topside since it has presented a good agreement between the modeled and theoretical scale height^19^. The semi-Epstein formulation is an analytical expression that represents the topside electron density by an exponential decay with increasing altitude. The equation is defined starting from the F2-layer peak density $N_m$ and the electron density is exponentially reduced as a function of the reduced height (*h* - $h_m$):

1 $$\begin{aligned} N_e = 4 N_m \frac{\exp {\frac{h-h_m}{H_s}}}{\left[1+\exp {\frac{h-h_m}{H_s}}\right]^2} \end{aligned}$$

The peak values $N_m$ and $h_m$ are typically measured by the RO techniques, topside sounders and ionosondes, so we have a long historical dataset to represent their values with a high fidelity. The scale height, on the other hand, is not directly measured by the instruments and its correct representation still remains under continuous investigation. To estimate the scale height and represent it with a linear height dependence up to  800 km, the $H_s$ values of the semi-Epstein function is firstly obtained with the following formulation^19^:

2 $$\begin{aligned} H_s = \frac{h-h_m}{\ln [ \frac{1}{N_e}(2N_m-N_e+2\sqrt{N_m^2 - N_e N_m}) ]} \end{aligned}$$

where the $N_e$ measurements are obtained by instruments capable to measure the topside ionosphere, such as topside sounders or GNSS-RO technologies.

Then, the scale height from Eq. (2) is fitted to accommodate to the following expression:

3 $$\begin{aligned} H_s^{linear}=H_0+\frac{\partial H_s}{\partial h}(h-h_m) \end{aligned}$$

where the electron density $N_e$ at the height *h* can be computed by knowing values from the electron density at the peak $N_m$, the peak height $h_m$ and the effective scale height $H_s$.

## The non-linear trend of the scale height at the plasmasphere

**Figure 1:**
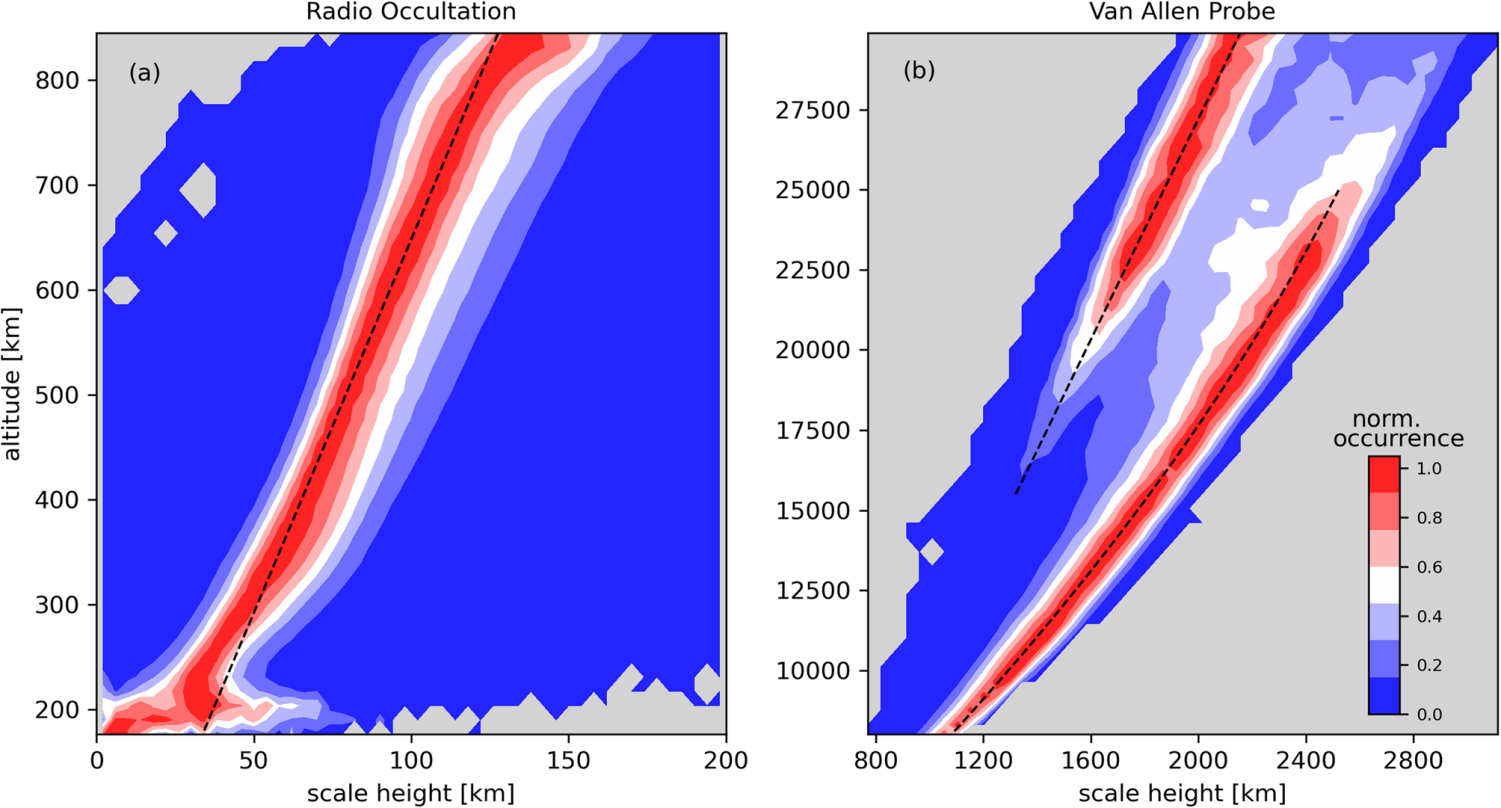
Altitude distribution of scale height values derived from: (**a**) the COSMIC RO measurements binned with 4 km in scale height and 13 km in altitude during the first 60 days of 2014 and (**b**) Van Allen Probe data using the entire dataset of 2014 binned with 44 km in scale height and 436 km in altitude. The color bar shows the normalized occurrence of the most predominant patterns of the dataset. Black dashed lines represent a linear fit on figures a) and b) and quadratic fit on figure b).

The linear trend of the scale height has been well investigated by previous studies. To demonstrate that RO electron density profiles can be well described by a linear dependence, Fig. 1a shows the general patterns of the scale height observed by ionospheric RO profiles from COSMIC. The distribution shows the number of occurrences of a specific $H_s$ value divided by the maximum number of $H_s$ occurrences of the specific height. The graph can be understood as two-dimensional histograms, where measurements of the first 60 days of 2014 were included in the analysis. The red color shows the maximum number of occurrences of $H_s$ for each specific height. The dashed black line shows a linear fitting of the scale height, presenting a great agreement above  250 km.

Previously, Prol et al.^18^ have detected a non-linear trend on the effective scale height when studying the ISIS and Allouette data, which covers the altitude range from the peak height up to around 2500 km. Such work has found that a quadratic scale height ($H_s^{quad}$) should benefit the electron density representation, being:

4 $$\begin{aligned} H_s^{quad}= H_0+ \frac{\partial H_s}{\partial h}(h-h_m)+\frac{1}{2} \frac{\partial ^2 H_s}{\partial h^2}(h-h_m)^2 \end{aligned}$$

where the term $\partial ^2 H_s/\partial h^2$ stands for the second partial derivative of the scale height.

The question that still remains is up to what extent the quadratic function should hold. To answer this point, Fig. 1b shows the main predominant patterns of the plasmasphere scale height retrieved from the Van Allen Probes $N_e$ measurements. We have applied Eq. (2) to the estimated scale height values based on Van Allen Probes electron densities retrieved from the NURD algorithm. The Van Allen Probe measurements relate to satellite path in the plasmasphere mostly at the equatorial region. Individual $N_e$ values along the satellite orbit are calculated from the upper hybrid resonant frequencies retrieved from the electric power spectral density spectrograms of the NURD algorithm (for details, see^37^). We can therefore estimate the corresponding $H_s$ value of each measurement and retrieve scale height distributions within the heights. The Van Allen Probes observations do not include measurements of the F2-peak height. In this regard, we have obtained peak values from the Neustrelitz Electron Density Model (NEDM2020)^30^.

In Fig. 1b, there are two distinct structures corresponding to high values of normalized occurrences. At altitudes from 8000 km up to  25,000 km, scale height increases from 1000 km up to approximately 2500 km, and the dependence can be well approximated using a quadratic trend line. Furthermore, at around 20,000 km altitude, one can observe an appearance of a second maximum of normalised occurrences with generally lower scale heights. The existence of this dual structure in scale height can be explained as follows. Ionospheric ion outflow along the geomagnetic field lines and form a region of cold plasma known as the plasmasphere. The outer boundary of the plasmasphere, known as the plasmapause, is characterized by a strong drop in electron density by approximately an order of magnitude. The plasmapause location is known to be highly dynamic (e.g.^41^), as under geomagnetically quiet conditions, it can expand up to 7 Earth radii over equator, while strongly contracting down to 2 Earth radii during geomagnetic storms^42^. The region outside of the plasmapause corresponds to much lower electron densities and is referred to as the plasmatrough. In Fig. 1b, the peaks of normalised occurrences at lower altitudes correspond to the plasmasphere, while the secondary maxima at higher altitudes are located in the plasmatrough. Due to the fact that the plasmapause location changes based on the geomagnetic conditions, on the averaged histogram shown in Fig. 1b there is an overlap region between the two populations. In order to separate scale height values corresponding to the plasmasphere and the plasmatrough, we use a criterion defined by Sheeley et al.^43^. The authors defined a boundary value of electron density as a function of L-shell, given by $n_b=10(6.6/L)^4$ el/cm$^3$. The values above this threshold are considered to be within the plasmasphere, while densities below the $n_b$ correspond to the plasmatrough. In this study, the scale height dependence on altitude was fitted separately for the two regions, and it was found that the plasmaspheric scale heights exhibit a quadratic trend, while the trend appears linear in the plasmatrough.

## On the link between RO and Van Allen Probe ionospheric profiles

**Figure 2:**
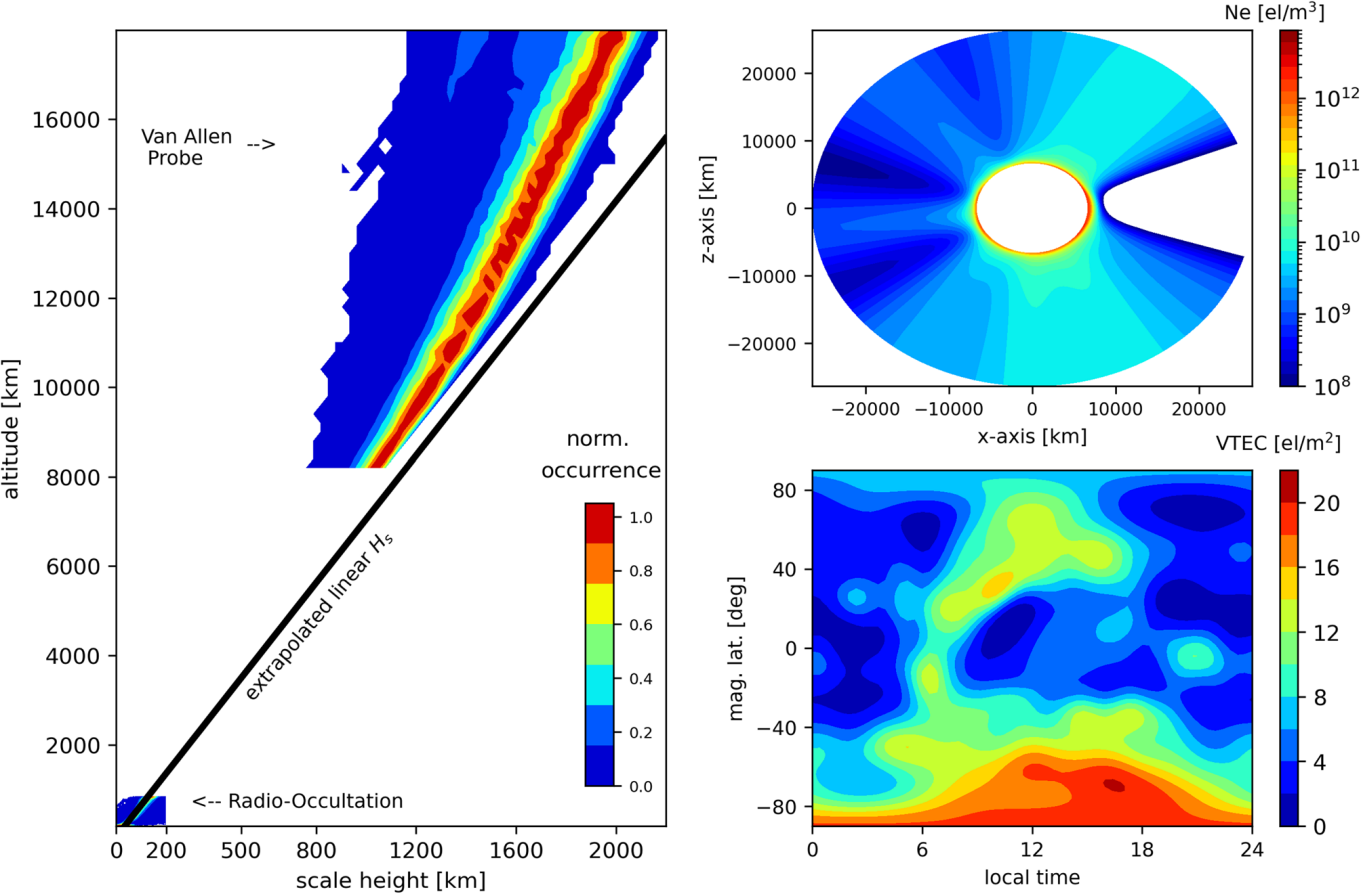
Left panel: 2D histogram of the normalized occurrence of the electron density distributions obtained by RO and Van Allen Probe. It shows the superposition of the graphs of Fig. 1, where the black line shows the $H_s$ linear extrapolation using RO measurements. Top right: Meridional cross-sections corresponding to the longitudinal section. One side is 0 and the opposite side is 180 E at zero hours in Universal Time (UT). The electron density profiles are linearly extrapolated with the RO measurements. Bottom right: VTEC representation from 800 km up to 20,000 km when the scale height is linearly extrapolated with the RO measurements. The RO data was obtained using averaged values from the first 60 days of 2014 using the harmonic fitting of Prol et al.^18^ in terms of local time and magnetic latitude.

In the previous section, we have shown that the Semi-Epstein function with quadratic $H_s$ dependence on altitude can be used to describe the electron density collected by Van Allen Probes at the plasmasphere. A point not yet answered, however, is how to connect the distributions of RO measurements to the Van Allen Probe data. An intuitive first guess would be to linearly extrapolate the RO scale height up to the plasmasphere. However, as demonstrated in Fig. 2, the extrapolation of the linear scale height given by RO measurements does not closely connect to the Van Allen Probe scale heights. In Fig. 2, left panel, the obtained linear fitting given by the RO measurements (up to  800 km) were extrapolated up to 20,000 km, presenting a significant overestimation of the black line for the altitudes above 8000 km. Moreover, the top-right panel shows expected meridional cross-sections of the electron density profiles when using the extrapolated linear $H_s$. The bottom panel shows the corresponding VTEC distribution computed from 800 km up to the GNSS height. Based on such results, we can clearly observe a misspecification of the extrapolated scale height. The electron density distributions do not follow the natural exponential decrease along the altitudes, as well as it does not produce expected VTEC maps. At the equatorial and low-latitude regions, the electron density strongly reduces within the altitudes, leading to almost absent electron densities and very low VTEC values. At middle and high-latitude regions, the electron density remains almost constant over the altitudes, providing unrealistic high VTEC values. Therefore, we can see that the linear trend observed by RO ionospheric profiles should not be directly extrapolated up to the plasmasphere.

**Figure 3:**
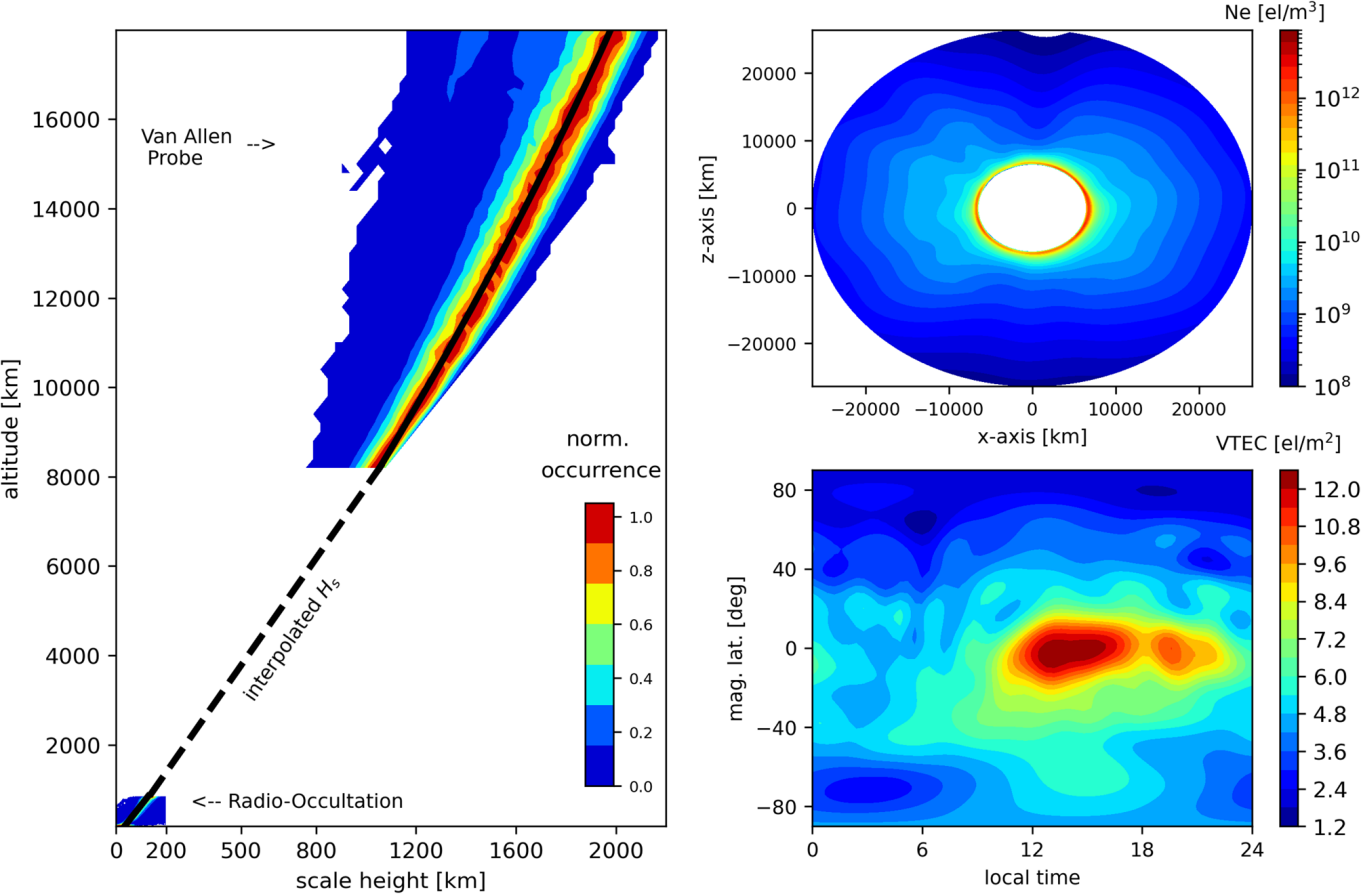
Same as Fig. 2, but using an interpolated $H_s$ at the transition region and Van Allen Probes $H_s$ at the plasmasphere.

To better link RO and Van Allen Probes, it appears to be practical to consider the ionosphere and plasmasphere regions as independent layers. The ionospheric layer obtained by RO measurements can be assumed from $h_m$ up to  800 km. The plasmaspheric layer observed by Van Allen Probes in our experiment covers altitudes above 7500 km. The gap between both layers is defined here as a transition region. Such assumption is consistent with first principles corresponding to the first layer with a denser O+ ion population near the F2-peak and a second layer associated with light ions, such as H+ and He+, which are the dominant ion species in the plasmasphere. As previously demonstrated, the topside ionospheric layer can be well described by a linear scale height. In the case of the plasmasphere, it seems reasonable to assume the scale height with quadratic $H_s$ dependence on altitude. Then, a model is necessary to properly connect the missing information between 800 to 7500 km. In the present study, the task to connect the ionospheric and plasmaspheric scale heights in the transition region is similar to the problem of connecting two separate lines. In this regard, the transition region can be represented by a simple linear function, i.e., a linear function connects the ionospheric $H_s$ at 800 km to the plasmaspheric $H_s$ at 7500 km, which allows to produce a smooth transition region. Figure 3 has the same format as Fig. 2, but now using the linear interpolation to represent the transition region in addition to the quadratic scale height in the plasmasphere. Now, we can observe expected meridional cross-sections and VTEC distributions of the plasmasphere, which gives an indication that RO electron densities can be merged with Van Allen Probe data to represent the plasmasphere.

## Combined model and its assessment

As discussed above, for describing the topside ionosphere and their assessments, we have identified that a Semi-Epstein function seems suitable to connect the RO ionospheric electron density to the Van Allen Probe plasmaspheric data. To further quantify how the use of this approximation can improve the fitting, this section provide a comparison between external VTEC data and the obtained electron density with the Semi-Epstein function. For the VTEC validation, we have developed a global model using RO and Van Allen Probe data. The global model is based on all key parameters needed to describe the electron density given by the Semi-Epstein function. A spherical harmonic function is used to describe each parameter globally in time and space given by the following equation:

5 $$\begin{aligned} f(\phi _m,t_L) = \sum _{n=0}^{N}\sum _{m=0}^{n} P_{nm}(\sin \phi _m) \left[\alpha _{nm} \cos (\pi \frac{m t_L}{12}) + \beta _{nm} \sin (\pi \frac{m t_L}{12})\right] \end{aligned}$$

where $f(\phi _m,t_L)$ stands for the observations on any of the target parameters, $\phi _m$ is the magnetic latitude, $t_L$ is the local time, $P_{nm}$ is the normalized associated Legendre polynomial, and $\alpha _{nm}$, and $\beta _{nm}$ are the coefficients to be determined in a least square adjustment with the degree N=10. The target parameters are related to the values needed to compute the electron density by Eq. (1). The two first parameters are related to the electron density at the peak $N_m$ and the peak height $h_m$, which are derived from RO observations, and the remaining parameters are related to the scale heights $H_s$.

In case of the ionospheric layer, a linear function is used, i.e., the scale height referred to the peak $H_0^I$ and the gradient of the scale height $\partial H_s^I/\partial h$ are estimated. The global distributions of the RO scale heights $H_0^I$ and $\partial H_s^I/\partial h$ is well depicted by Prol et al.^18^. In case of the plasmaspheric layer, in addition to $H_0^P$ and $\partial H_s^P/\partial h$ referred to the plasmasphere, the second derivative of the scale height $\partial ^2 H_s^P/\partial h^2$ is estimated. The plasma trough is removed from the Van Allen Probes dataset to better determine the plasmasphere decay up to 20,000 km. Since most of the Van Allen Probes electron densities are measured near the geomagnetic equator, a cosine dependence with the magnetic latitude was included in the estimations^44^. Additionally, the transition layer is described by $H_0^T$, $\partial H_s^T/\partial h$, which connects the ionospheric layer to the plasmasphere with a linear scale height.

Despite several years of COSMIC data are available to be used in the modelling process, Van Allen Probe data is rather limited to cover entire solar cycles in the estimation process. In this regard, we use monthly data averages to represent the plasmasphere and topside ionosphere. A previous investigation by Prol et al.^17^ has shown that 60 consecutive days of superimposed RO data, that is a time-window of 30 days, can provide global coverage without the need of large datasets. Additionally, Prol et al.^45^ have proven that the referred approach provides a great background model for ionospheric tomography, and it has compared well against ionosonde data.

**Figure 4:**
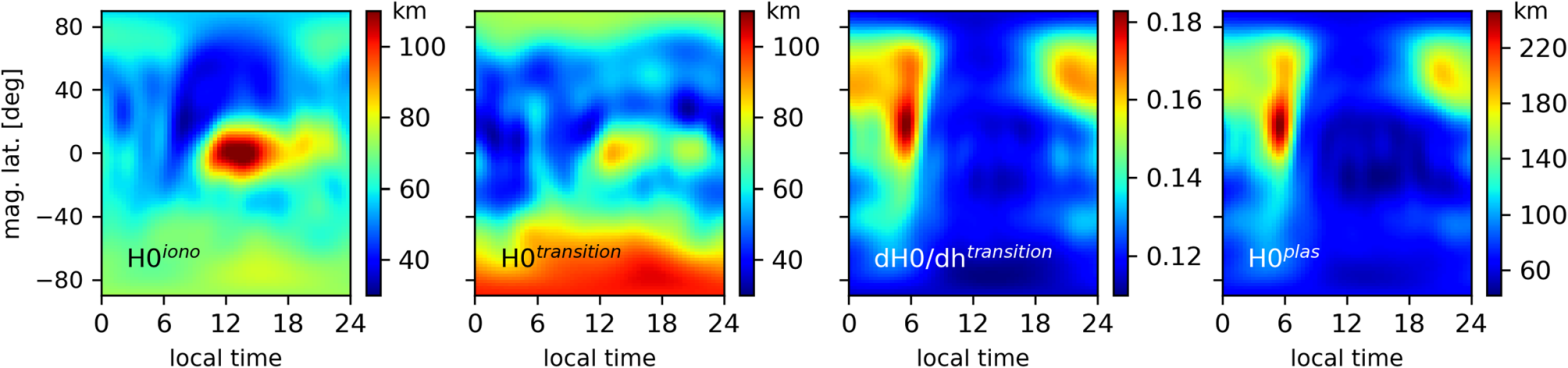
Scale height estimations for the ionosphere ($H0^{iono}$), transition region ($H0^{transition}$ and $\partial H0/\partial h^{transition}$) and plasmasphere ($H0^{plas}$) in terms of local time by magnetic latitude. The representations were obtained using RO and Van Allen Probes data of the first 60 days of 2014, i.e., during the Southern Summer.

From our investigations, Fig. 4 shows the most predominant patterns of the transition region. The scale height of the transition region is similar to the scale height of the ionosphere. The gradient of the transition region, however, accommodates better to the plasmaspheric scale height. Therefore, the transition region acts as an intermediate layer to smoothly change from the ionosphere to the plasmasphere distributions. As for the plasmaspheric scale height $H_0^P$, the distributions do not directly reflect the plasmasphere variability. As previously mentioned, the plasmaspheric profiles are anchored to the ionospheric peak height $h_m$. Any variation of $h_m$ or $N_m$ impacts the $H_0^P$ fitting estimation, in a way that the estimated electron density in the plasmasphere accommodates to the Van Allen Probe data. As a consequence, Fig. 4 shows how much the scale height needs to adapt to connect the $h_m$ and $N_m$ variations given by RO data to the Van Allen Probe electron densities so that the representations reflect the different horizontal patterns between the ionosphere and plasmasphere. As it can be seen, there are signatures of the equatorial ionization anomaly (EIA) in the plasmaspheric scale height, which do exist in RO data, but not in plasmaspheric distributions. Contrarily, there are large values in the nighttime scale height ( 22 to 06 LT), which occurs due to the larger ionization of the plasmasphere in comparison to the ionosphere. The reason for this nighttime increase has been first discussed in Prol et al.^17^, which points out to be related to the local sunrise. At the local sunrise, the sunlight first reaches the plasmasphere. The increasing of the ion production in the upper part of the topside raises the electron density, but no significant variation occurs at the ionospheric peak height.

**Figure 5:**
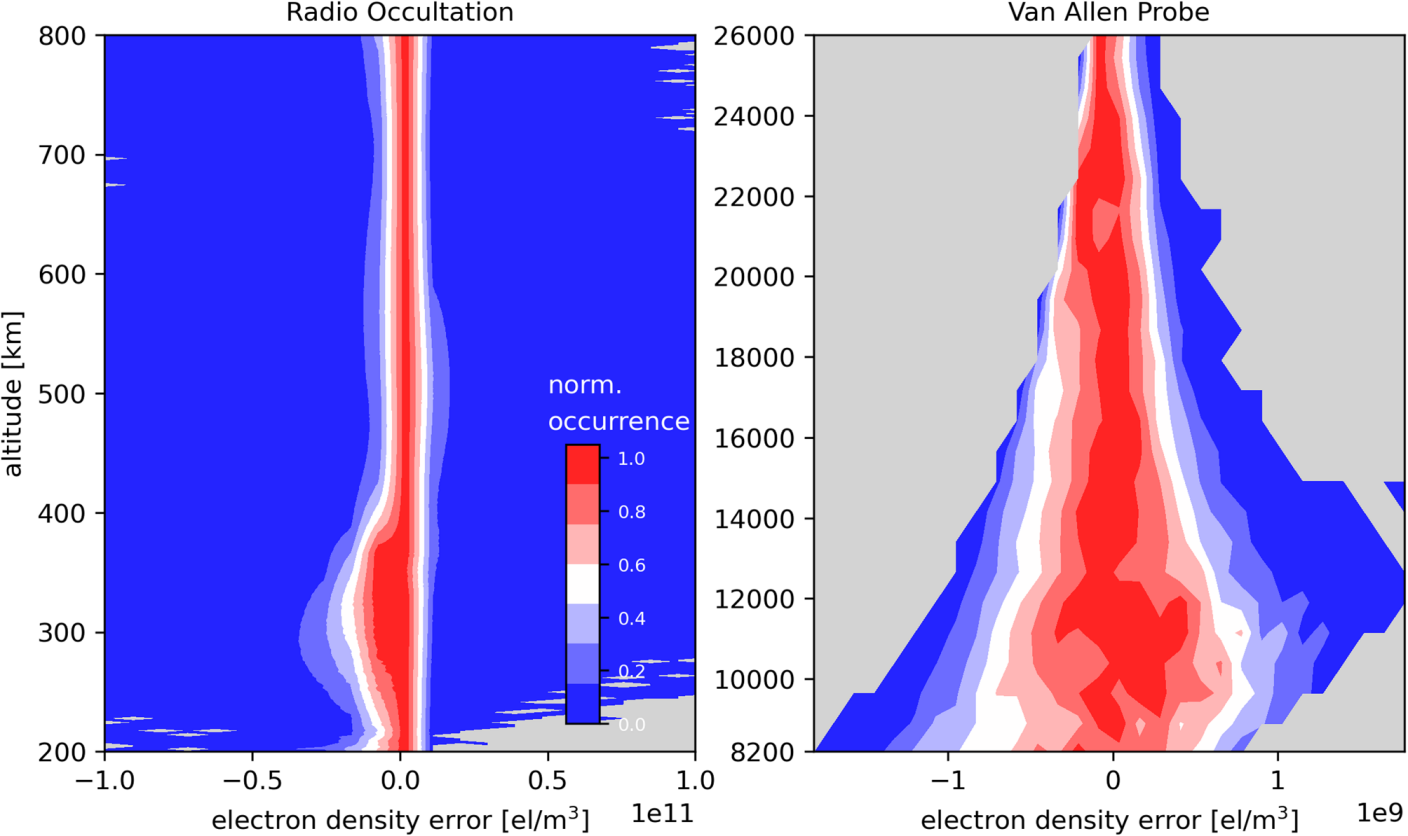
Distributions of the $N_e$ discrepancies between: the developed method and RO measurements (left panel); the developed method and Van Allen Probes data (right panel).

An evaluation of the model’s performance is first conducted to compare the estimated electron density with measurements during the first 60 days of 2014 (high solar activity). Figure 5 (left panel) shows 2D histograms of the electron density discrepancies between the modeled values and the actual RO observations. The right panel shows the same, but for the Van Allen Probes. Both errors distributions are centered at zero, which means that the profile function together with the spherical harmonic model well accommodates to the actual measurements, providing no significant bias. Therefore, the proposed function can be used to describe general patterns of the exponential electron density decay within the altitudes. The computed mean error is -6.04*$10^8$ and -0.11*$10^8$ el/m$^3$ for the RO and Van Allen Probes datasets, respectively. The standard deviation of the error is 137.46*$10^8$ and 2.76*$10^8$ el/m$^3$ for the RO and Van Allen Probe datasets, respectively, showing a better performance on the plasmasphere.

**Figure 6:**
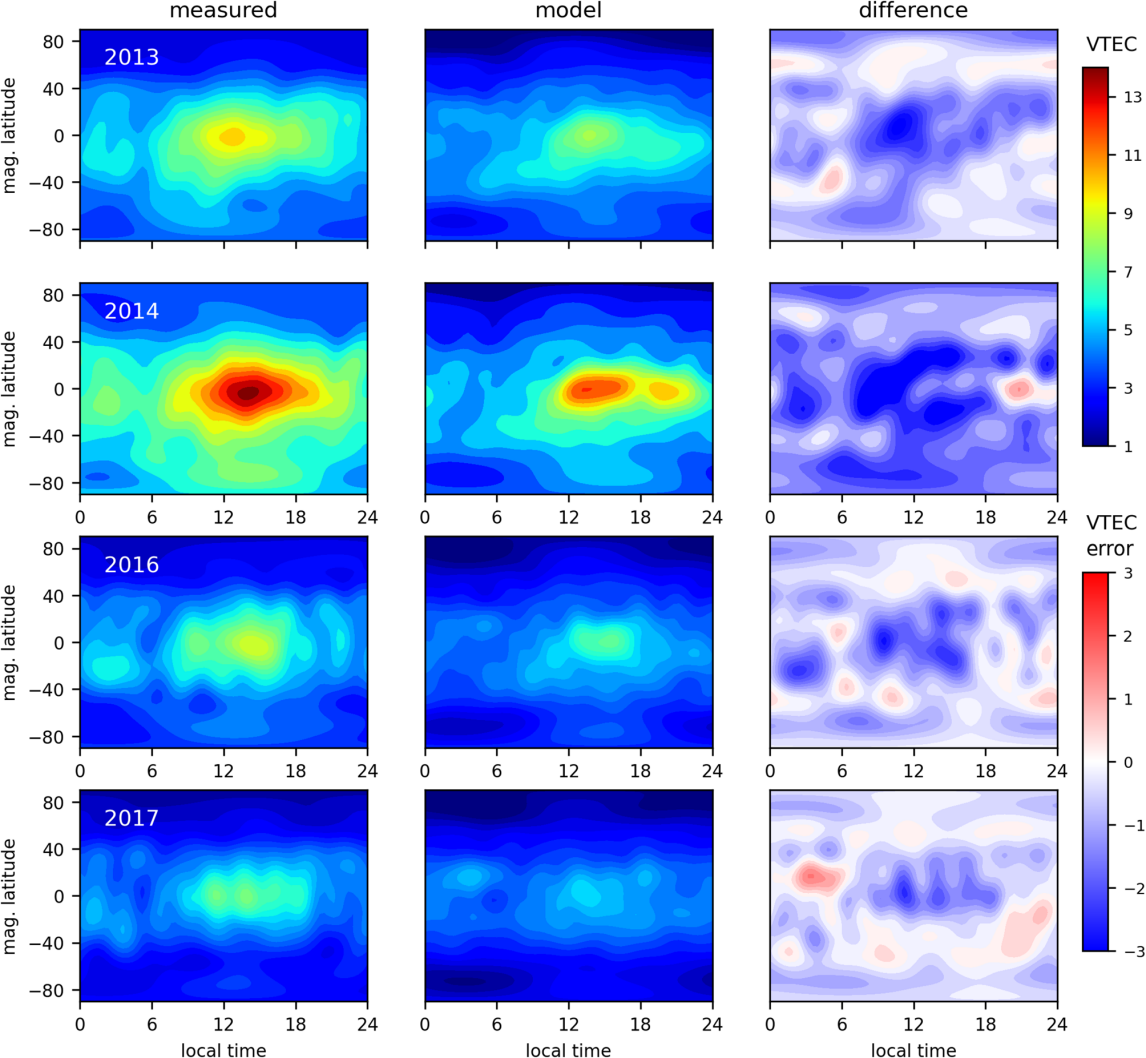
Topside VTEC maps in terms of local time and magnetic latitude obtained by actual TEC observations of COSMIC (left) and the proposed function using RO and Van Allen Probe observations (center). The right graph shows the discrepancies of the estimations. Top panels are related to 2013 and bottom panels are related to 2017. The unit of the color bar is TECU.

The evaluation of the function developed in terms of external VTEC data is carried out using podTEC values. All computations were performed to represent actual COSMIC measurements of the topside TEC. The slant TEC was converted to the vertical direction (VTEC) using the mapping function proposed by Foelsche and Kirchengast^46^ with a single-shell height of 1300 km, which has been shown as one of the best heights to represent podTEC values of COSMIC^47^. Figure 6 shows the obtained VTEC distributions in terms of local time and magnetic latitude related to the days of the year 1 to 60 in four years. Each map represents sixty days of data superimposition in order to cover the entire globe with observations. Top panels are related to 2013 and bottom panels are related to 2017. As we can see, the function developed is capable to represent the main distributions of the topside VTEC over the distinct years. It can be seen that maximum TEC values are observed around 14:00 hours LT (local time) in the equatorial region. The VTEC then diminishes towards to the nighttime and the polar region. Since all periods are related to the Southern Summer, there is a higher ionization level in the Southern hemisphere, which was captured by the developed fit. The average error of the results is -0.8, -1.6, -0.7, -0.4 TECU for 2013, 2014, 2016 and 2017, respectively. The standard deviation of the error is 0.6, 0.7, 0.6, 0.5 TECU for 2013, 2014, 2016 and 2017, respectively. There is still room for improvement related to the underestimation in the daytime equatorial region. We believe this underestimation occurs due to a few possible reasons. As shown by Smirnov et al.^48^, COSMIC radio-occultation measurements slightly underestimate the electron density in comparison to the Gravity Recovery and Climate Experiment (GRACE) data, mainly at the EIA crests, which is exactly where we found the strongest underestimations in Fig. 6, 2014. Another potential reason for this underestimation is the missing information within the transition region, which is known to have complex dynamics, including formation of electron holes at altitudes of around 1500-3000 km (e.g.,^49^), which may not be well-captured due to the altitudinal data gaps between the RO and Van Allen Probes data used in this experiment. In addition, the maximum error of 3 TECU (TEC Units), where 1 TECU=$10^{16}$ el/m$^2$, is expected^38^ since the COSMIC TEC accuracy is around 1 to 3 TECU due to leveling errors, multipath and differential code bias calibration. Thus, it is reasonable to say that using RO together with Van Allen Probes data provides a suitable representation of the upper ionosphere and plasmasphere not only in the profile shape but also in the VTEC magnitude and distribution, with a slightly underestimation of around 1 TECU.

## Conclusions

This study has presented evidence that Van Allen Probe electron density measurements can be combined with RO COSMIC observations to describe the topside ionosphere and plasmasphere. Our investigation has demonstrated that the linear dependence of the scale height cannot be directly used to extrapolate ionospehric RO profiles above 800 km. Indeed, above 7500 km, a quadratic pattern was found to be more predominant. We then propose a multi-layer semi-Epstein function to describe the scale height by means of linear and quadratic dependences. Results have shown that the proposed function is capable to capture the main patterns from the peak height up to 20,000 km and well describe the electron density decay within the altitudes at the ionosphere and plasmasphere. The proposed model was also capable to represent the main distributions of the topside VTEC based on podTEC measurements for periods of low and high solar activities. This suggests that we can rely on the profile representation given by the quadratic functions, not only in the profile shape representation but also in the VTEC magnitude and distributions. Thus, it is reasonable to say that the Semi-Epstein function using RO together with Van Allen Probes provides a suitable representation of the upper ionosphere and plasmasphere. This is an important finding since traditional empirical models, such as NeQuick and the IRI-Plas, need to be essentially improved above the F2 layer peak (hmF2) for better characterization of the topside TEC^33^. Additionally, the knowledge of plasmaspheric electron content to account for the electron content above the LEO satellite orbit height is necessary for improved ionospheric radio occultation inversion. In the future, it would be relevant to check whether the proposed model can improve the accuracy of the RO electron density inversion. It is also recommended to develop better strategies to represent the transition region.

## Data Availability

The datasets generated during the current study are available from the corresponding author on reasonable request. The raw GNSS dataset can be obtained through the COSMIC Data Analysis and Archive Center (CDAAC) via the portal https://cdaac-www.cosmic.ucar.edu/. The Van Allen Probes obtained by the NURD dataset is available at https://dataservices.gfz-potsdam.de/.
